# A fabricated hydrogel of hyaluronic acid/curcumin shows super-activity to heal the bacterial infected wound

**DOI:** 10.1186/s13568-023-01533-y

**Published:** 2023-03-10

**Authors:** Maryam Khaleghi, Fakhri Haghi, Mina Gholami, Hamdam Hourfar, Farshad Shahi, Ali Mir Mousavi Zekoloujeh, Farhang Aliakbari, Ebrahim Ahmadi, Dina Morshedi

**Affiliations:** 1 Bioprocess Engineering Department, Institute of Industrial and Environmental Biotechnology, National Institute of Genetic Engineering and Biotechnology, Shahrak-E Pajoohesh, km 15 Tehran-Karaj Highway, 14965/161, Tehran, Iran; 2 Department of Microbiology, School of Medicine, Zanjan University of Medical Sciences, 45139-56111 Zanjan, IR Iran; 3 Department of Biology, University of Zanjan, Zanjan, Iran; 4 Molecular Medicine Research Group, Robarts Research Institute, Schulich School of Medicine and Dentistry, University of Western Ontario, London, ON Canada; 5 Department of Chemistry, University of Zanjan, Zanjan, Iran

**Keywords:** Antibacterial hydrogel, Curcumin, Hyaluronic acid, Scarless, Skin regeneration, Wound healing

## Abstract

High risk of acute morbidities and even mortality from expanding the antibiotics resistant infectious wounds force indefinite efforts for development of high performance wound-healing materials. Herein, we design a procedure to fabricate a hyaluronic acid (HA)-based hydrogel to conjugate curcumin (Gel-H.P.Cur). The highlight of this work is to provide a favorite condition for capturing curcumin while protecting its structure and intensifying its activities because of the synchronization with HA. Accordingly, HA as a major component of dermis with a critical role in establishing skin health, could fortify the wound healing property as well as antibacterial activity of the hydrogel. Gel-H.P.Cur showed antibacterial properties against *Pseudomonas aeruginosa* (*P. aeruginosa*), which were examined by bactericidal efficiency, disk diffusion, anti-biofilm, and pyocyanin production assays. The effects of Gel-H.P.Cur on the inhibition of quorum sensing (QS) regulatory genes that contribute to expanding bacteria in the injured place was also significant. In addition, Gel-H.P.Cur showed high potential to heal the cutaneous wounds on the mouse excisional wound model with repairing histopathological damages rapidly and without scar. Taken together, the results strongly support Gel-H.P.Cur as a multipotent biomaterial for medical applications regarding the treatment of chronic, infected, and dehiscent wounds.

**Supplementary Information:**

The online version contains supplementary material available at 10.1186/s13568-023-01533-y.

## Introduction

Skin is the largest organ in the body and, as a natural barrier, protects humans from pathogens, physicochemical harms, and losing the body’s fluid. Every year, millions of people worldwide suffer from many acute and chronic skin disorders, especially due to impaired wound healing or failing to treat the resistant infectious disease (Wild et al. 2010).

Wound healing is a complex phenomenon, including inflammation, proliferation, and remodeling, which finally leads to skin regeneration (Sushma et al. 2018). The main threat during wound healing is infection with different pathogenic and opportunistic microorganisms such as *Pseudomonas aeruginosa* (*P.* *aeruginosa*), *Staphylococcus aureus* (*S. aureus*)*,* and *Escherichia coli* (*E. coli*) (Qureshi et al. 2015). Different medicinal approaches have been recommended to protect the injured skin, such as wound dressings (Chen et al. 2018), however, traditional wound dressings (gauze, bandage etc.) has some shortcomings such as low capacity for the wound exudate adsorption, strong adhesion and skin-stripping after frequent replacement of the dressing (Lawton and Langøen 2009, Qureshi et al. 2015). Wound exudates contain proteases that induce more damages in the surrounding epidermis and subsequently prolong the healing process, as well as promote the growth of alkalophilic microorganisms (Greener et al. 2005). Modern medicine tries to develop new strategies for wound dressing by using new gentle materials like hydrogels. While hydrogels have high capacity for absorbing wound exudates, they can provide the essential moisture for the wound healing process (Zhao et al. 2017), protect skin against invasive microorganisms, afford appropriate dynamicity at the treated site, and good permeability to oxygen with its porous structure (Qu et al. 2019).

As a natural polymer, hyaluronic acid (HA) has gained increasing attention to be used in the therapeutic hydrogels, especially for wound dressing because of its biocompatibility, biodegradability, high capacity for adsorption of aqueous solutions, and its natural role in the healing process (Jeong et al. 2018, Frenkel 2014). In addition, the role of HA against microorganisms, particularly its anti-adhesion and anti-biofilm properties, has been of interest (Romanò et al. 2017). Nonetheless, poor mechanical strength in the hydrated state limits the biomedical applications of HA (Jeong et al. 2018). Cross-linking with appropriate cross-linkers is one of the strategies to improve HA’s mechanical properties. Cross-linkers are small and multi-functional molecules that can react with the functional groups of HA (hydroxyl, carboxylic acid, or amino groups) (Additional file 1: Fig. S1A) (Khaleghi et al. 2020). Using pre-functionalized polymers is another strategy that eliminates the use of cross-linkers. Polydimethylsiloxane-diglycidyl ether terminated (PDMS-DG) with epoxy group at its ends (bis-epoxide PDMS) (Additional file 1: Fig. S1B), is a non-toxic, biocompatible, gas permeable, transparent, and flexible polymer that is used to fabricate bio-medical devices (Halldorsson et al. 2015).

It believes that overuse of antibiotics, in addition to antibiotic resistance, can be toxic for the vulnerable and sensitive skin cells growing around the wound and delay the wound healing process. Plants are sources of the safe and affordable remedial compounds that stimulate the healing process (Qureshi et al. 2015). One of the most used herbal compounds in traditional and modern medicine is curcumin or diferuloylmethane [1, 7-bis (4-hydroxy-3-methoxyphenyl)-1, 6-heptadiene-3, 5-dione] with numerous pharmacological effects, including antioxidant, anti-inflammatory, anti-mutagenic, anti-cancer and anti-amyloid fibrillation activities (Tafvizizavareh et al. 2019; Hewlings and Kalman 2017). Curcumin is a natural small molecule belonging to the group of polyphenolic curcuminoids, with a bright yellow color. Curcumin is confronted with a wide range of microbes, including bacteria (both Gram-positive and Gram-negative), viruses, and also fungi (Zorofchian Moghadamtousi et al. 2014). Curcumin is a valuable candidate to use for wound healing because it is safe even at high doses (12 gr/day) and used as a FDA approved drug widely (Zorofchian Moghadamtousi et al. 2014; Hewlings and Kalman 2017). However, due to its low solubility and stability in the free form at physiological condition, some strategies such as using nano carriers, encapsulating within colloidal particles and synthesizing its some derivatives/conjugates have been employed thus far (Zheng and McClements 2020; Taebnia et al. 2016).

In the present study, in order to develop a curcumin-supplied hyaluronic based hydrogel for wound healing purposes we cross-linked PDMS-DG to HA via epoxy-OH mediated polymer–polymer reaction (Khaleghi et al. 2020) and then we successfully ameliorated the wound healing properties of the hydrogel because of loading high amount of curcumin in its native form. Next to analyzing the physicochemical characters of the curcumin-loaded hydrogel (Gel-H.P.Cur), its antibacterial and wound healing potential was examined using the in vitro and in vivo methods.

## Experimental section

### Materials

Curcumin was purchased from Acros organic (Geel, Belgium). Polydimethylsiloxane-diglycidyl ether terminated (epoxy terminated PDMS or PDMS-DG, 480282-50ML, average Mn ~ 800, Japan), DMSO and all other chemicals were from Sigma-Aldrich. Dry powder of sodium hyaluronate (average molecular weight = 50000 Da) was purchased from BulkActives (Taiwan).

### Fabrication of the curcumin-loaded hydrogel (Gel-H.P.Cur)

To produce the curcumin-loaded hydrogel (Gel-H.P.Cur), at the first step, the HA-PDMS hydrogel was produced as discussed previously in detail with some modifications (Khaleghi et al. 2020). 100 mg (0.128 mmol) of HA powder dissolved in 0.5 mL distilled water plus 50 µL of NaOH (0.2 N, pH > 10). In another container, the PDMS solution was prepared by adding 200 µL of DMSO and 50 µL of NaOH (0.2 N, pH > 10) to 1 mL of PDMS-DG (1.23 mmol) and mixed thoroughly by mechanical agitation for 60 s and then left for 30 min. After that, the PDMS solution was added to the HA solution, stirred for 120 s, and the mixture incubated at 37 °C for 2 h. After removing the non-reacted part, the resulting hydrogel (Gel-H.P) was kept at 25 °C with an open door for 48 h. Then, 0.5 mL of curcumin solution (10 mg/mL, dissolved in DMSO) was added to the semi-dried hydrogel and the mixture was incubated at room temperature (RT) till no more curcumin adsorbed (measuring the absorbance of solution). Finally, the immersed hydrogel containing curcumin (Gel-H.P.Cur) was stored at RT for further analysis. To ascertain whether DMSO plays a role in the antibacterial effect of Gel-H.P.Cur, semi dried Gel-H.P was immersed in 0.5 mL of DMSO (without curcumin) for 48 h (Gel-H.P.DMSO). Then the Gel-H.P.DMSO was stored at RT for antibacterial assay.

### Characterization of the hydrogels

#### Nuclear magnetic resonance spectroscopy (NMR)

Gel-H.P dialyzed for 24 h against distilled water and then lyophilized. The lyophilized hydrogel dissolved in D_2_O and was applied for NMR analysis using a Bruker Avance 250 MHZ spectrometer. Gel-H.P.Cur was immersed in DMSO for 48 h and then the NMR of released curcumin in DMSO was studied.The NMR data was processed using MestReNova software.

#### Fourier-transformed infrared (FTIR) and attenuated total reflectance (ATR) FTIR analyses

FTIR spectrum of curcumin was recorded using a Nicolet iS 10 FTIR spectrometer after preparing the sample using the KBr pellet method. ATR-FTIR spectra of Gel-H.P and Gel-H.P.Cur were obtained using the ATR (Bruker, Tensor27, Equinox55). All spectra were recorded at 1 cm^−1^ resolution in the range of 600–4000 cm^−1^.

#### Scanning electron microscopy (SEM)

The microstructure of the gels (Gel-H.P and Gel-H.P.Cur) were compared using a TESCAN mira2 (the Czech Republic) SEM, according to the following steps: first, the hydrogels were fully swollen in distilled water, then the hydrogels were lyophilized and immediately coated with gold.

#### Swelling index determination

The pre-weighted (Wd) dried hydrogel (Gel-H.P.Cur) was immersed in distilled water at 37 °C and at predetermined time intervals, its swollen form was weighted (Ws) after removing the excess water using tissue paper. Swelling measurement was continued until no difference was observed between two consecutive Ws. The degree of swelling obtained using Eq. (1):

1 $${\text{Degree of swelling }}\left( {\text{\% }} \right) = \frac{{{\text{Ws }} - {\text{Wd}}}}{{{\text{Wd}}}}{ } \times 100$$

### Antibacterial activity assays

#### Bacterial strain and its growth condition

*P. aeruginosa* PAO1 as the wild type strain was donated from the department of microbiology, school of medicine, Zanjan University of medical science. Cultivation of *P. aeruginosa* was carried out using Mueller Hinton (MH) or Luria Bertani (LB) broth/agar at 37 °C. *P. aeruginosa* was stocked in trypticase soy broth (TSB) containing 20% (*v/v*) glycerol and stored at − 70 °C for further studies.

#### Assessing the bactericidal efficiency of the fabricated hydrogels by dilution and disc diffusion methods

Cell growth inhibitory effect of Gel-H.P, Gel-H.P.Cur and Gel-H.P.DMSO against *P. aeruginosa* was investigated using the broth dilution method, according to the Clinical and Laboratory Standards Institute (CLSI) guidelines (CLSI 2015). Briefly, the ODs of the cultures were recorded at 620 nm (LKB Biochrom 4050, Ultraspec II UV/Vis spectrophotometer) after treating *P. aeruginosa* (1.5 × 10^5^ colony-forming unit (CFU)/mL) with different concentrations (0.078–5 mg/mL) of the sterilized (by UV irradiation for 30 min) Gel-H.P, Gel-H.P.Cur or Gel-H.P.DMSO for 18 h at 37 °C. The bactericidal activities of the hydrogels were quantified using Eq. (2) (OD values were the mean of three measurements). Besides, the cultured bacteria without the hydrogels and the media without any bacteria were considered as positive and negative controls, respectively.

2 $${\text{Bactericidal activities \% }} = 1 - \frac{{{\text{OD}}620{ }\left( {\text{treated culture}} \right){ }}}{{{\text{OD}}620{ }\,\left( {\text{untreated culture}} \right)}}{ } \times 100$$

In the disc diffusion method,* P. aeruginosa* with inoculum concentrations of 10^8^ CFU/mL was spread on Mueller Hinton agar petri-dish, and a piece of each hydrogel (5 mg) was placed on the plate. After incubation for 18 h at 37 °C, the zone of growth inhibition was measured using a ruler (Imipenem disc (10 μg, Mast Group Ltd., Merseyside, UK), was used as standard control).

#### Assessing the biofilm formation

The effects of Gel-H.P and Gel-H.P.Cur on the biofilm formation of *P. aeruginosa* monitored by the microtitre plate based on crystal violet assay (Noshiranzadeh et al. 2017). The overnight cultures incubated with different concentrations (2.5, 5, and 10 mg/mL) of Gel-H.P and Gel-H.P.Cur at 37 °C for 18 h (in order to prepare different concentrations of hydrogels, solid hydrogels were dissolved in MH medium). After removing free planktonic cells, the plates were washed three times with phosphate-buffered saline (PBS) and fixed with 150 µL of methanol. The plates were stained with crystal violet (0.5% w/v) for 10 min. After washing off the excess dye with distilled water, glacial acetic acid was added and incubated for 20 min to solubilize crystal violet. Finally, the absorbance was read at 570 nm in an absorbance microplate reader (Biotek microplate reader ELx-808, USA). Bacteria without hydrogels and the media without any bacteria were considered as positive and negative controls, respectively. OD values were the mean of three measurements that converted to % of inhibition of biofilm formation by Eq. (3).

3 $${\text{\% of inhibition of biofilm formation }} = 1 - \frac{{{\text{A}}570{\text{ of treated PAO}}1}}{{{\text{A}}570{\text{ of untreated PAO}}1}}{ } \times 100$$

#### Effects of the synthesized hydrogels on the pyocyanin production by *P. aeruginosa*

Pyocyanin production as a virulence factor of *P. aeruginosa* was measured based on the method described previously (Heidari et al. 2017). In this regard, 500 µL of bacteria (1 × 10^8^ CFU/mL) were cultured in the presence of Gel-H.P.Cur (2.5, 5, 10 mg/mL) or Gel-H.P (10 mg/mL) in a final volume of 10 mL (LB broth medium) at 37 °C for 24 h. To extract pyocyanin, 3 mL chloroform was mixed with 5 mL of the supernatant, after centrifugation of the culture at 8000 rpm for 5 min. After forming two phases, the chloroform phase extracted and transferred to another test tube contained 1 mL HCl (0.2 M). The solution was centrifuged at 12,000 rpm at 4 °C for 10 min and then the absorbance of the supernatant was recorded at 520 nm. The final concentration of pyocyanin was calculated by Eq. (4) (Absorbance values were the mean of three measurements).

4 $$\left[ {{\text{Pyocyanin}}} \right] \, = {\text{ A52}}0 \, \times { 17}.0{7}$$

### Quorum sensing (QS) regulatory genes expression analysis

#### RNA extraction and cDNA synthesis

At the first step using RNeasy Mini Kit (QIAGEN, Hilden, Germany), total RNA was extracted from PAO1 cultivated broth culture at the exponential growth phase in the presence of Gel-H.P, Gel-H.P.Cur (10 mg/mL) or alone (as control). The concentration and purity of the extracted RNA were evaluated with NanoDropTM ND-1000 spectrophotometer (Nano-Drop Technologies, Wilmington, DE) at 260 nm and 260/280 nm, respectively. To synthesis cDNA, we employed a High-Capacity cDNA Reverse Transcription Kit (Applied Biosystems, Foster City, CA). Reverse transcription (RT) was performed in a reaction mixture with a total volume of 20 mL containing 10 mL of RNA (800 ng), 2 mL of RT buffer (10x), 0.8 mL of deoxynucleoside triphosphate (25x), 2 mL of RT random primers (100 mM) and 1 mL of reverse transcriptase (1 U). The reactions were incubated at 25 °C for 10 min, at 37 °C for 120 min, at 85 °C for 5 min and at 4 °C for 10 min.

#### Real-time quantitative PCR (qPCR)

To explore the effect of Gel-H.P and Gel-H.P.Cur (10 mg/mL) on the expression of QS circuit genes including *lasI*, *lasR*, *rhlI* and *rhlR*, we carried out real-time qPCR using the primers listed in Additional file 1: Table S1 (Bahari et al. 2017).

Each primer was mixed with 10 mL of 2 × SYBR Green PCR Master Mix (Exiqon, Denmark). Assays performed by a Rotor Gene-6000 real-time analyzer (Corbett Research, Qiagen) in triplicate. All data were normalized to the internal standard *oprL* (encoding the outer membrane protein OprL), and the melting curve analysis demonstrated that the accumulation of SYBR Green-bound DNA was target gene-specific. The negative control with no treatment was included in the experiments. To determine the threshold cycle values (C_t_), the C_t_ for amplification of each gene was normalized to the C_t_ of the amplified *oprL*’s gene in the related sample. Then, ΔC_t_ values were compared with the control without any treatment based on Eq. (5).

5 $$\begin{gathered} \Delta {\text{Ct }}\,{\text{sample }} = {\text{ Ct }}\,{\text{sample}}\, - {\text{Ct }}\,\,{\text{oprL}}\,\,{\text{ sample}} \hfill \\ \Delta {\text{Ct control }} = {\text{ Ct }}\,{\text{control}} - \,{\text{Ct oprL}}\,{\text{ control}} \hfill \\ \end{gathered}$$

### Animal model studies

#### Wound closure

The wound healing capabilities of Gel-H.P and Gel-H.P.Cur were evaluated using a mouse excisional wound model (BALB/c). Six-eight week-old male mice were randomly divided into 6 groups (Three mice in each group) (Table 1); Group1: treated only with PBS (100 µL), Group 2: treated with Gel-H.P, Group 3: treated with Gel-H.P.Cur, Group 4: infected with 100 µL of *P. aeruginosa* (0.5 McFarland) without any treatment, Group 5: infected with 100 µL of *P. aeruginosa* (0.5 McFarland) and also treated with Gel-H.P, Group 6: infected with 100 µL of *P. aeruginosa* (0.5 McFarland) and also treated with Gel-H.P.Cur. Treatment was performed with 100 µL of the hydrogels (10 mg/mL) dissolved in PBS. After anesthetization deeply by injection of a mixture of ketamine (50 mg/kg of body weight) and xylazine (5 mg/kg of body weight), the dorsum of the mice were shaved, disinfected using 70% ethanol solution, and punched (in diameter of 15 mm) by employing sterile scissors. Treatments (using PBS, Gel-H.P, Gel-H.P.Cur, or *P. aeruginosa*) applied only on the day of surgery and did not repeat over the next 15 days. On the 3rd, 6th, 9th, 12th, and 15th days after wounding, we took photographs of wounds by a digital camera. The percentage of the wound closure was calculated by Eq. (6), in which Ai and At are initial wound area and wound area each day, respectively. The size of the wound area was calculated using ImageJ software.

6 $${\text{Wound closure }}\left( {\text{\% }} \right) = \frac{{\left( {{\text{Ai }}{-}{\text{ At}}} \right)}}{{\text{Ai }}}{ } \times { }100$$

**Table 1:** Details of each group of incision wound model mice

Number	Group	Treatment agent	Infection
1	PBS	PBS	–
2	Gel-H.P	Gel-H.P	–
3	Gel-H.P.Cur	Gel-H.P.Cur	–
4	PAO1	–	+
5	PAO1 + Gel-H.P	Gel-H.P	+
6	PAO1 + Gel-H.P.Cur	Gel-H.P.Cur	+

Finally, on the day 15th, mice were sacrificed for histopathological analysis.

The animal studies carried out here, were approved by the National Institutes of Genetic Engineering and Biotechnology (NIGEB) Animal Care Committee (IR.NIGEB.EC.1399.4.9.D).

#### Histopathological study

For preparation of the histological slides, the wound sites and the surrounding areas (groups 4 and 6, Table 1) were excised and fixed in 10% formalin. The biopsies were embedded in paraffin wax, sectioned by microtome (Leica, RM, 2135, USA), and stained with hematoxylin and eosin (H&E) according to the standard method (Shefa et al. 2020, 2017).

### Statistical analysis

All experiments were carried out in triplicate, and the results presented as means ± SD. The statistical significance within the groups was analyzed using One-way ANOVA. The significance outcome between the groups was also computed using unpaired Student’s t-test (P value < 0.05 was considered significant).

## Results

### Appropriate conditions for the fabrication of Gel-H.P with curcumin (Gel-H.P.Cur)

**Fig. 1:**
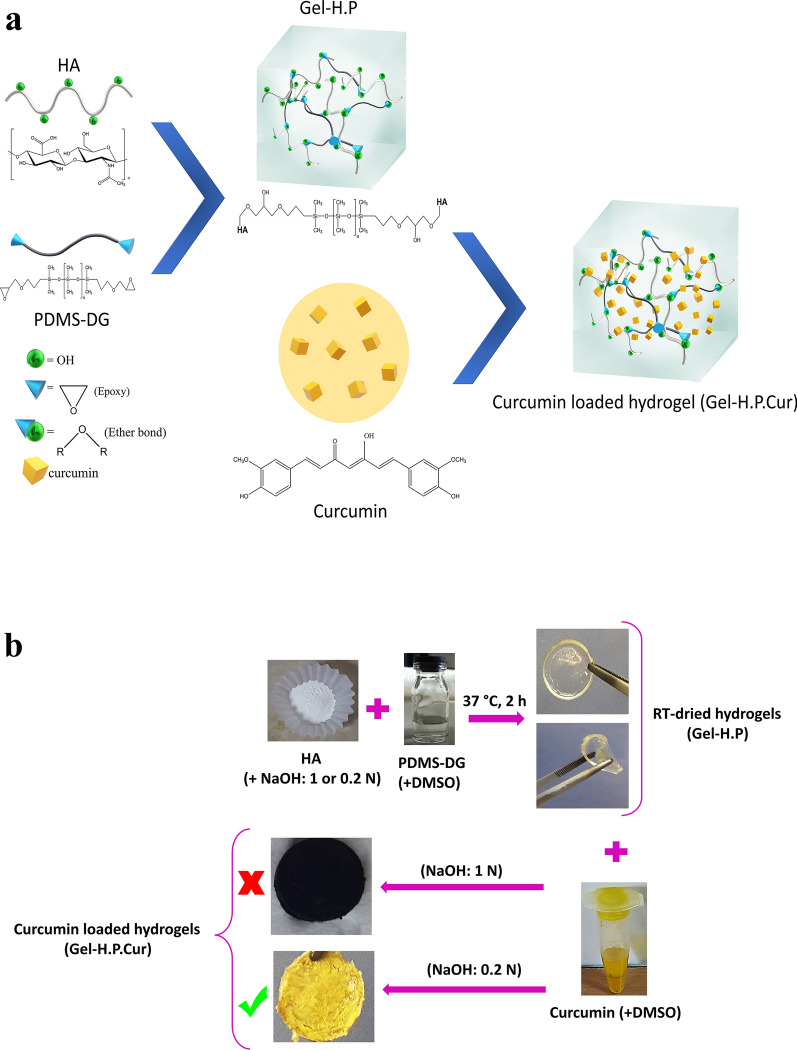
Synthesis of Gel-H.P and Gel-H.P.Cur. **a** Schematic and **b** Original materials representation. HA was incubated at basic pH, and the interaction of epoxide groups with the HA hydroxyl groups leads to ether bond formation. This cross-linking reaction created the HA-PDMS 3D hydrogel network. By adsorbing and penetrating curcumin in the Gel-H.P (at appropriate pH condition) the color completely converted to yellow even in the inner parts of Gel-H.P.Cur

At the first step, we synthesized a stable, biocompatible porous hydrogel composed of HA and PDMS-DG based on our recently developed method with slight modifications which was addressed in the method section (Khaleghi et al. 2020). After soaking the semi-dried hydrogel in the curcumin solution (10 mg/mL, dissolved in DMSO), curcumin absorbed and then permeated into the inner parts of the Gel-H.P, which was accompanied by turning the color of the hydrogel uniformly to yellow (Gel-H.P.Cur). Figure 1a, b schematically shows synthesis steps of Gel-H.P.Cur which consist of the final product in the adequate and inadequate conditions. Curcumin undergoes severe discoloration (brown to black) when exposed to alkaline conditions and is prone to structural degradation (Kharat et al. 2017; Kumavat et al. 2013). Therefore, in this study, the conditions leading to the black hydrogel withdrew and the analyses were followed by the yellow hydrogel.

**Fig. 2:**
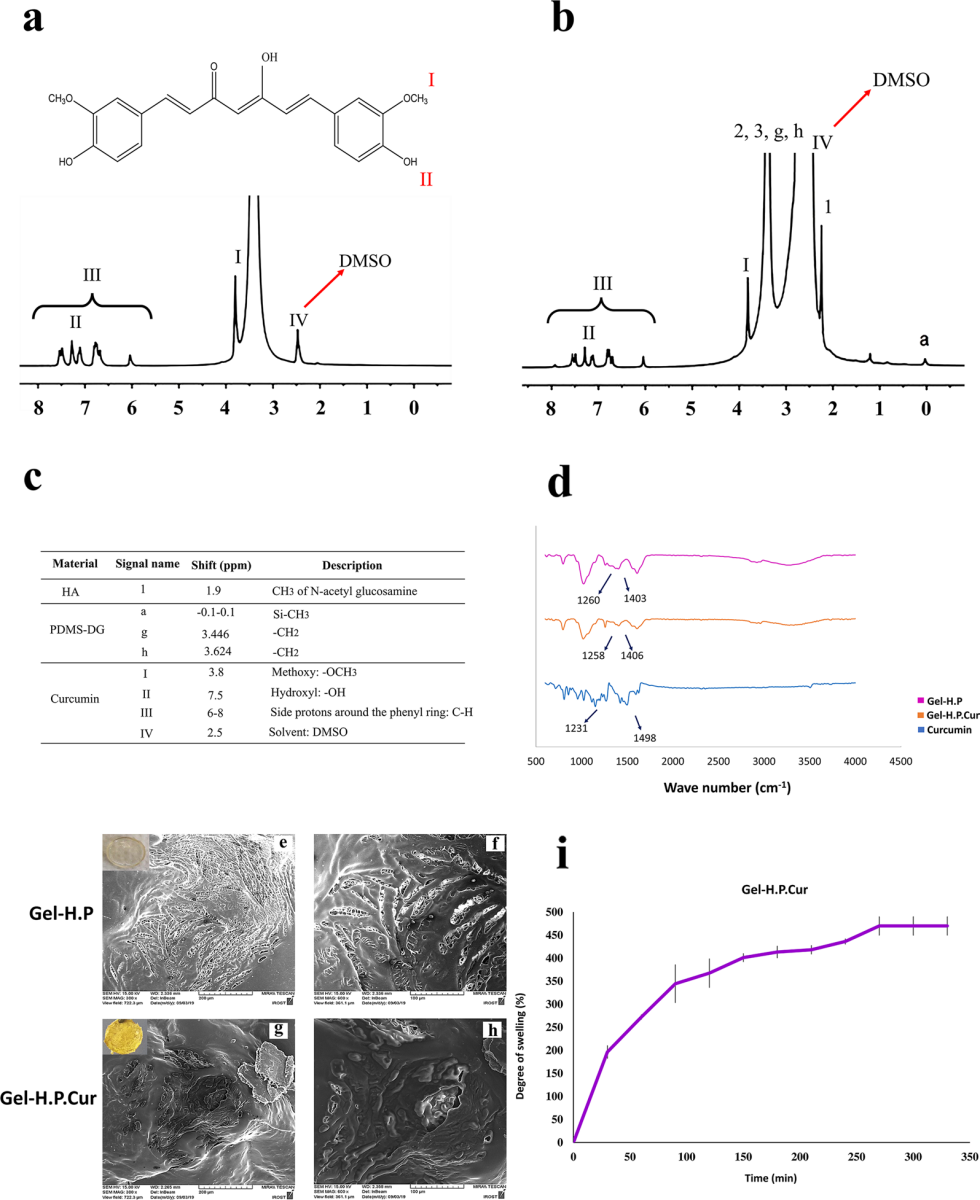
Hydrogels characterization using NMR, FTIR, SEM and swelling assay. **a** ^1^H NMR spectrum of the pure curcumin in DMSO with specific peaks for the methoxy group (I), hydroxyl group (II) and other protons around the phenyl ring (II). **b** ^1^H NMR spectrum of the curcumin released from Gel-H.P.Cur with specific peaks of pure curcumin (I, II, III). Traces of hyaluronic acid (1: CH_3_ of N-acetyl glucosamine and 2, 3: protons around the sugar ring) and PDMS (a: Si-CH_3_: and g, h: CH_2_) were detected in the spectrum. The red numbers in the chemical structure represented the chemical groups detected by NMR. **c** NMR characteristic peaks of pure curcumin and the curcumin released from Gel-H.P.Cur. **d** FTIR spectra of Gel-H.P, Gel-H.P.Cur, and Curcumin. Characteristic peaks of HA (~ 1400 cm^−1^ related to carboxylic acid: O–H bend), PDMS-DG (~ 1260 cm^−1^ related to Si–C) and curcumin (1231 cm^−1^: phenolic C–O group and 1498 cm^−1^: aromatic C=O band stretching vibration) were detected. **e**–**h** SEM micrographs of the hydrogels: **e** and **f** are micrographs for Gel-H.P, and **g** and **h** are for Gel-H.P.Cur. Imaging was performed after coating the lyophilized hydrogels with gold. The detail of magnification and scale bars are demonstrated under each image. **i** Assessment of the swelling rate of the Gel-H.P.Cur in distilled water at 37 °C. Gel-H.P.Cur showed a high level of water absorption

NMR, FTIR, SEM as well as swelling measurements were employed to identify the properties of the fabricated Gel-H.P.Cur. NMR data for Gel-H.P has been reported previously; however, a summary of the characteristic peaks of NMR has shown in Additional file 1: Table S2 (Khaleghi et al. 2020). Moreover, to declare that curcumin remained in its native form during the synthesis process, the NMR spectra of pure curcumin and the released curcumin from the hydrogel were analyzed (Fig. 2a, b). In NMR spectra, we also detected some traces of hyaluronic acid (CH_3_ of n-acetyl glucosamine, 1.9 ppm) and PDMS-DG (Si-CH_3_, -0.1–0.1 ppm) (Fig. 2b, c).

FTIR data showed the typical peaks of HA and PDMS-DG in both hydrogels (~ 1400 cm^−1^ related to carboxylic acid: O–H bend and ~ 1260 cm^−1^ related to Si–C) (Fig. 2d). The curcumin characteristic peaks have also appeared in 1231 cm^−1^: phenolic C–O group and 1498 cm^−1^: aromatic C=O band stretching vibration.

To verify the microstructure of the hydrogels, SEM imaging was also carried out (Fig. 2e–h). Gel-H.P (Fig. 2e, f) showed a more porous structure than Gel-H.P.Cur (Fig. 2g, h). However, the rate of swelling with absorbing more than 469% (w/w) of water during 330 min indicated that the fabricated composite including curcumin capable to uptake high amount of aqueous materials (Fig. 2i).

### Gel-H.P.Cur exhibited inhibitory effects on the growth of P. aeruginosa

**Fig. 3:**
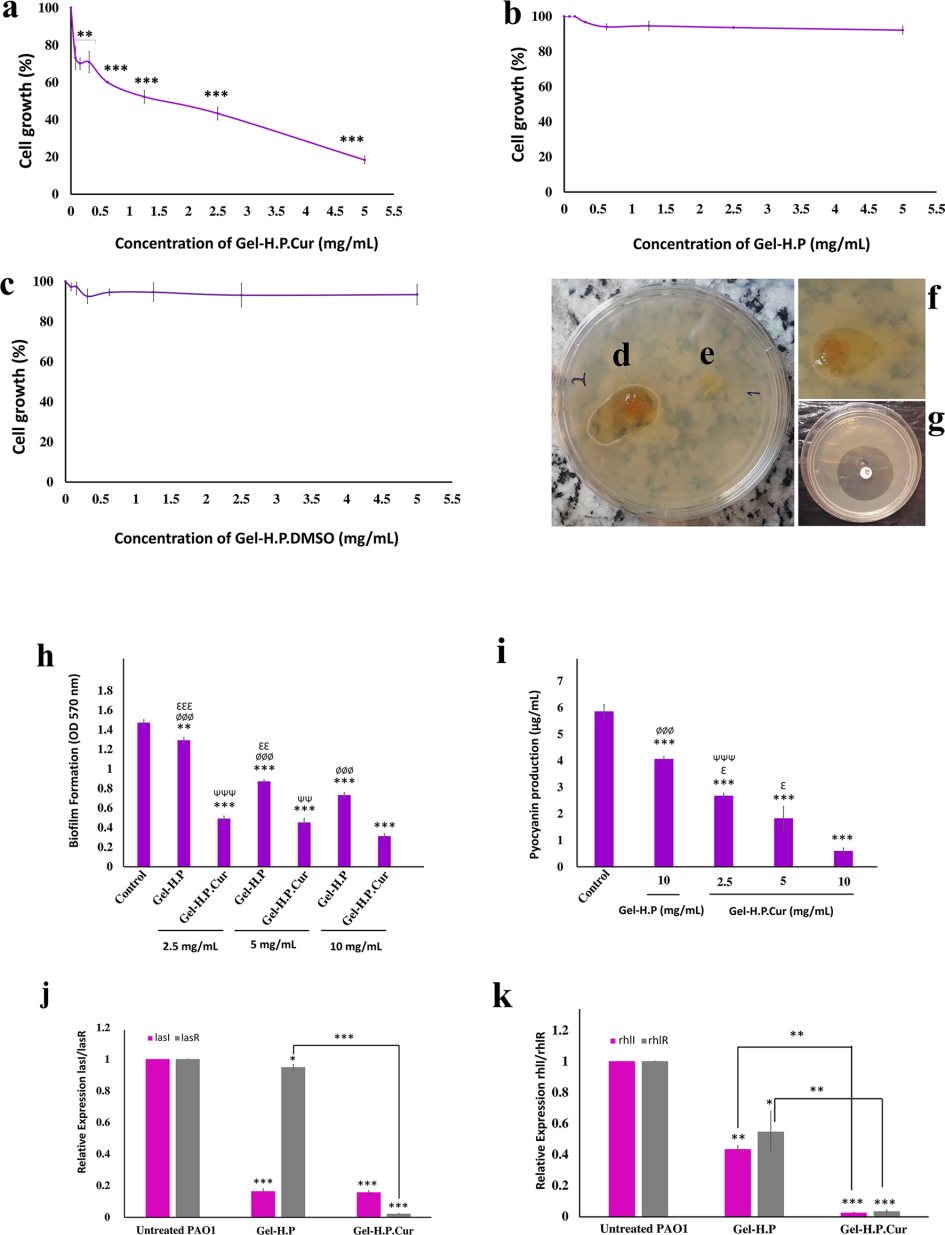
In vitro study of antibacterial activity of the hydrogels. **a**–**c** Serial dilution method: Inhibitory effect of different concentrations of Gel-H.P.Cur, Gel-H.P and Gel-H.P.DMSO on *P. aeruginosa* growth in MH broth during 18 h at 37 °C (mean ± SD, * P < 0.05, ** P < 0.01, *** P < 0.001). (**d**–**g**) Disc diffusion method: The zone formation appeared from the growth inhibition around Gel-H.P.Cur (**d** and **f**) and imipenem disc (**g**) but not Gel-H.P (**e**) on MH agar plate. The zone of growth inhibition was measured (cm.^2^) and recorded using a ruler and a digital camera, respectively. **h** Assessment of *P. aeruginosa* biofilm formation in the presence of the hydrogels, using crystal violet staining standard method. Inhibitory effect of different concentrations (2.5, 5 and 10 mg/mL) of Gel-H.P or Gel-H.P.Cur on biofilm formation of *P. aeruginosa* was assayed after 18 h incubation in MH broth at 37 °C. *: statistically significant differences between treated and control groups. Ø: sstatistically significant differences between Gel-H.P and Gel-H.P.Cur with the same concentration (2.5, 5 or 10 mg/mL). Ɛ: statistically significant differences between Gel-H.P (2.5 mg/mL) and Gel-H.P (5 mg/mL), Gel-H.P (2.5 mg/mL) and Gel-H.P (10 mg/mL), Gel-H.P (5 mg/mL) and Gel-H.P (10 mg/mL). Ψ: statistically significant differences between Gel-H.P.Cur (2.5 mg/mL) and Gel-H.P.Cur (10 mg/mL), Gel-H.P.Cur (5 mg/mL) and Gel-H.P.Cur (10 mg/mL). (Mean ± SD, **, ƐƐ, ΨΨ P < 0.01, ***, ØØØ, ƐƐƐ, ΨΨΨ P < 0.001). (i) Inhibitory effect of different concentrations of Gel-H.P (10 mg/mL) or Gel-H.P.Cur (2.5, 5 and 10 mg/mL) on pyocyanin production of *P. aeruginosa* during 24 h incubation in LB broth at 37 °C. *: statistically significant differences between treated and control groups. Ø: statistically significant differences between Gel-H.P (10 mg/mL) and Gel-H.P.Cur (with different concentrations: 2.5, 5 or 10 mg/mL). Ɛ: statistically significant differences between Gel-H.P.Cur (2.5 mg/mL) and Gel-H.P.Cur (5 mg/mL), Gel-H.P.Cur (5 mg/mL) and Gel-H.P.Cur (10 mg/mL). Ψ: statistically significant differences between Gel-H.P.Cur (2.5 mg/mL) and Gel-H.P.Cur (10 mg/mL). (Mean ± SD, Ɛ P < 0.05, ***, ØØØ, ΨΨΨ P < 0.001). (j and k) Effect of hydrogels (Gel-H.P and Gel-H.P.Cur) at a concentration of 10 mg/mL on the expression level of quorum sensing (QS) regulatory genes (f: *lasI/lasR,* g:* rhlI/rhlR*) in *P. aeruginosa* during 12 h incubation. Both hydrogels significantly reduced the expression of QS genes compared to the control (untreated) group (mean ± SD, * P < 0.05, ** P < 0.01, *** P < 0.001)

We determined whether there was a significant reduction in the growth rate of *P. aeruginosa* when treated with Gel-H.P.Cur (treatment with 0.078–5 mg/mL of Gel-H.P.Cur: Reduction of bacteria from the concentration of 16.8 × 10^8^ CFU/mL to 12.3–3.1 × 10^8^ CFU/mL) (Fig. 3a). Note that Gel-H.P and Gel-H.P.DMSO showed no significant inhibitory effect against *P. aeruginosa* (Fig. 3b, c). We observed about a 2.62 ± 0.59 cm^2^ zone of growth inhibition in the disk diffusion test around the Gel-H.P.Cur on MHA plate pre-covered with *P. aeruginosa* (Fig. 3d and f) but not for Gel-H.P (Fig. 3e). These results could reflect the diffusion ability of curcumin in a solid surface even after its entrapping through the fabricated hydrogel. The zone of growth inhibition produced by the imipenem disc, as a standard control, was about 3.5 cm^2^ (Fig. 3g).

### Biofilm formation, Pyocyanin production as well as the Quorum Sensing (QS) regulatory genes expression in P. aeruginosa decreased by both hydrogels (Gel-H.P and Gel-H.P.Cur)

Both hydrogels (Gel-H.P and Gel-H.P.Cur) decreased the biofilm formation remarkably (Fig. 3h). However, in the presence of curcumin the anti-biofilm effect obviously increased which could reflect the synergistic impacts of HA and curcumin.

Pyocyanin is a non-fluorescent blue-green pigment produced by *P. aeruginosa*, which has an important role in the infectious potential of *P. aeruginosa* (Vinckx 2010). Both hydrogels significantly reduced pyocyanin production with a maximum reduction of 89.92% and 30.6% for Gel-H.P.Cur and Gel-H.P, respectively (Fig. 3i).

Quorum sensing (QS) is a cell-to-cell signaling system that controls virulence factors, antibiotic resistance, and biofilm formation in bacteria by regulating gene expression in response to the cell density (Bassler and Losick 2006; El-Mowafy et al. 2014). *Pseudomonas* has three distinct QS systems including *las*, *rhl* and *MvfR* (PqsR), which are mediated by small signal molecules called autoinducers. The *lasI* and *rhlI* products, direct the synthesis of autoinducers called n- (3-oxo-dodecanoyl) -l-homoserin lactone (3-oxo-C12-AHL) and n- (butanoyl) -l-homoserine lactone (C4-AHL), respectively. These diffusible signaling molecules can interact with *lasR* and *rhlR* (respectively) to activate the target promoters (El-Mowafy et al. 2014, Venturi 2006). By using real-time PCR, the effects of Gel-H.P and Gel-H.P.Cur on the expression of QS regulatory genes including *lasI*/*lasR* and *rhlI*/*rhlR* were investigated. In the presence of the hydrogels, the expression of all genes was significantly reduced (Fig. 3j, k).

### Gel-H.P and Gel-H.P.Cur rebuilt the infected wounds

**Fig. 4:**
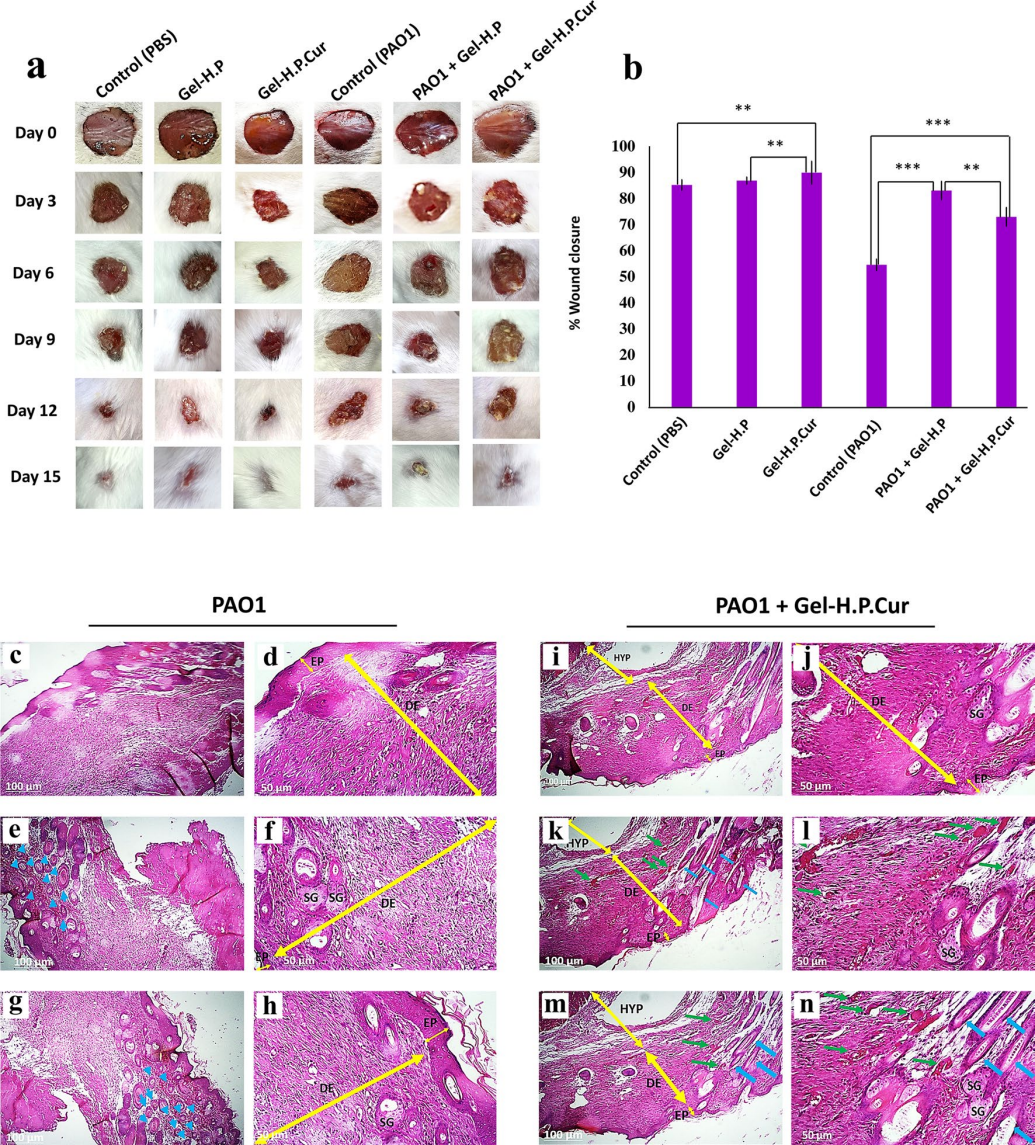
Macroscopic and microscopic photographs of wound healing. **a** Wound healing appearances in all control and treated (Gel-H.P or Gel-H.P.Cur/infected or non-infected) groups (Three mice in each group) on days 0, 3, 6, 9, 12 and 15 were recorded using a digital camera. All of the treatments were performed only once on wounding day (day 0) and the healing process was followed until day 15. **b** Wound closure rate (%) of 6 groups on the day 12 next to post wounding. Wound area was measured using Image J software and the ratio of the wound size on the 12th to the zero days was calculated (mean ± SD, * P < 0.05, ** P < 0.01, *** P < 0.001). **c**–**n** Histopathological evaluation of the wounds using H&E staining on day 15 post-wounding. **c**, **d**, **e**, **f**, **g** and **h** are the control group (Table 1, group 4, *P. aeruginosa*: infected with 100 µL of *P. aeruginosa* (0.5 McFarland) without any treatment), while **i**, **j**, **k**, **l**, **m** and **n** are the treated group (Table 1, group 6, PAO1 + Gel-H.P.Cur: infected with 100 µL of *P. aeruginosa* (0.5 McFarland) + treated with Gel H.P.Cur). *EP* epidermis, *DE* dermis, *HYP* hypodermis, *SG* sebaceous glands, yellow arrows: EP, DE or HYP, blue arrows: hair follicles, green arrows: blood vessels

To screen the wound healing activity of the synthesized hydrogels in an extensive skin lesions infected with *P. aeruginosa* as real samples, we used a full-thickness incision wound model in mice. The wound healing process followed for up to 2 weeks (Fig. 4a). Quantitative data on day 12th revealed that the group treated with Gel-H.P.Cur (without bacterial infection, group 3: Gel-H.P.Cur, Table 1) and the group infected with *P. aeruginosa* (without hydrogel application, group 4: PAO1, Table 1) had the highest (89.85% ± 4.35) and the lowest (54.51% ± 2.2) wound closure, respectively (Fig. 4b). Both HA and curcumin might have remediation roles on the wounds.

Histopathological analysis indicated that treatment with Gel-H.P.Cur improved the restoration of natural tissue structure in the injured skin on the day 15^th^ post-wounding (Fig. 4c–n). Epidermis (EP: yellow arrow) and dermis (DE: yellow arrow) were traceable in untreated and treated skin with Gel-H.P.Cur; however, hypodermis (low density tissue but contains a lot of fat) was only seen in treated skin (HYP: yellow arrow). Although some of cutaneous annexes such as sebaceous glands (SG) and many immature hair follicles (blue arrows) observed in the untreated skin, SG, adult hair follicles (blue arrows) and blood vessels (green arrows) were generated in the treated skin.

## Discussion

Turmeric is definitely one of the oldest herbal extracts with well-known anti-inflammatory, antimicrobial, antioxidant, chemopreventive and chemotherapeutic properties (Sharifi-Rad et al. 2020; Zorofchian Moghadamtousi et al. 2014). This traditional spice has been used in the treatment of various diseases in many Asian countries for thousands of years (Ammon and Wahl 1991). After the discovery of curcumin as the main active ingredient of turmeric, studies have shifted on its activities. For topical treatment of cutaneous wound healing however, combining curcumin with the materials that provide expanded surfaces, like bandages and films are highly recommended (Mohanty and Sahoo 2017). Also, hydrogels with characteristics such as porous three-dimensional structure, permeability to oxygen, swelling or liquid absorption, mechanical flexibility, and no secondary damage in the wound can be a suitable choice for the new wound dressings development (Liu et al. 2022). Recently, biomedical researchers have focused on the synthesis of antibacterial hydrogels to develop effective wound dressings (Rahmani et al. 2021; Shang et al. 2022). Therefore, in this study for the cutaneous wound healing purpose, we chose cross-linked HA hydrogel. We have already shown that applying an epoxy compound (Polydimethylsiloxane-diglycidyl ether terminated) provided a porous HA-based hydrogel with high rate of swelling (up to 500%), with a very good resistance versus enzymatic and chemical degradations (Khaleghi et al. 2020). However, during combination with curcumin, the color of curcumin converted to dark brown due to the fabrication condition. It is indicated that curcumin mostly is in its enolate form at alkaline condition which is in labile and unstable tautomeric form and its color rapidly converts from bright yellow to dark brown (Bhatia et al. 2016). By modifying the synthesis process of HA-PDMS hydrogel and condition for combining of curcumin, the color of the composite remains in light yellow color.

NMR and FTIR data confirmed that the structure of curcumin remained stable during the incorporating process. The specific peaks of curcumin including methoxy (− OCH_3_, 3.8 ppm), hydroxyl ( −OH, 7.5 ppm) and side protons around the phenyl ring (C–H, 6.6–8.4 ppm) were observed in the NMR spectra of free curcumin as well as when it released from Gel-H.P.Cur. Moreover, the FTIR spectra indicated the characteristic peaks of HA and PDMS-DG as well as curcumin. The data fortified the assumption that the incorporation of curcumin in Gel-H.P probably is a weak chemical or just physical adsorption with no considerable changes in the main peaks of the all three components. For our related purpose, it is better that the incorporated compound does not interact strongly with the hydrogel matrix and is easily released to the media. SEM micrographs showed the porous and net-like topology of the hydrogels. The pores of the Gel-H.P surface were much clearer and more regular, however, in the case of Gel-H.P.Cur, the pores were less regular which could be due to the filling with curcumin. It should be noted, while curcumin releases, the pores could absorb the wound secretions instead. It should be noted that the effect of the cross-linking process on the porosity and swelling properties of the hydrogel has been explained in detail in the other study (Khaleghi et al. 2020). High potential to trap the aqueous materials is another critical factor for wound dressings mainly in two respects including adsorption of the excess wound exudates and prevention of the secondary infections (Wahid et al. 2016; El-Kased et al. 2017). Besides, hydrogels with high swelling values (such as HA-based hydrogels) provide a hydrated microenvironment similar to natural milieu for arranging the migration of dermal cells (i.e., fibroblasts, keratinocytes, or endothelial cells) in the wound bed and subsequently inducing wound closure and the tissue regeneration (Zhu et al. 2006, Chen 2002). Furthermore, the capacity of hydrogels for entrapment of drugs directly relates to their swelling capability (Marulasiddeshwara et al. 2020). We determined that Gel-H.P.Cur had a high degree of swelling (up to 500% w/w) which was mostly similar to Gel-H.P’s swelling rate.

To examine the antibacterial activities of Gel-H.P and Gel-H.P.Cur in the in vitro models, *P. aeruginosa* was chosen, since it is one of the most abundant Gram-negative bacteria in the infections such as bloodstream infections, pneumonia, urinary tract infections, and skin infections especially surgical wound infections that can cause severe difficulties and even death, particularly in the immunosuppressed patients (Moore and Flaws 2011; Paprocka et al. 2022). Antibiotic selection for *P. aeruginosa* treatment is complicated and has become a major concern for the word-wide health issues because it involves many multidrug-resistant strains (Amini and Namvar 2019). In this study, we observed the inhibitory effect of Gel-H.P.Cur on the growth of *P. aeruginosa* using liquid culturing and disk diffusion methods, whereas we did not perceive the obvious effect of Gel-H.P on the growth of the bacteria. Disk diffusion method revealed that curcumin can diffuse in media from the fabricated hydrogel, however, according to some studies, it may also work even in its incorporated state (Taebnia et al. 2016). The antibacterial activity of HA/Cur composite has also been investigated in a recent study. Using disc diffusion and minimal inhibitory concentration assay methods, Snetkov et al. showed HA/Cur composite nanofibers have an inhibitory effect on a variety of microbial strains, especially Gram-positive bacteria (Snetkov et al. 2022).

Although Gel-H.P did not attenuate the growth of *P. aeruginosa* considerably, we saw that it could boost curcumin performances against some activities of *P. aeruginosa* such as biofilm formation, pyocyanin production, and the quorum sensing (QS) regulatory genes expressions, all of which are the pathogenicity and invasion factors in bacteria. The formation of biofilm causes bacterial resistance to the host immune system, antimicrobial agents, UV light exposure, chemical disinfectants, and temperature stress (Dastgheyb et al. 2015). Blocking or even disturbing biofilm formation may be a valuable and significant step in the treatment of microbial infection. In this study, Gel-H.P without curcumin showed anti-biofilm impact. The anti-biofilm activity of HA as the major part of Gel-H.P, has already been determined (Drago et al. 2014). There are some studies indicating that some wound healing dressings are prone to contamination (Lei et al. 2019). However, as an achievements of our study, no contamination of Gel-H.P.Cur for long time (7 months) was detected suggesting that the presence of curcumin may amplify anticontamination property of the fabricated hydrogel (Khaleghi et al. 2020).

We also determined the positive effect of Gel-H.P on the curcumin activities against production of pyocyanin by *P. aeruginosa*. Pyocyanin is a water-soluble greenish-yellow secondary metabolite that stimulates *P. aeruginosa* to resist humane immune responses (Ran et al. 2003, Vinckx , 2010). Curcumin has shown the inhibitory effect on the biosynthesis of pyocyanin. It has been shown that treatment with curcumin (1.5–3 µg/mL) reduced the pyocyanin production in *P. aeruginosa* by 80% (Rudrappa and Bais 2008). Here we found for the first time that Gel-H.P (without curcumin) significantly inhibited pyocyanin production in a dose dependent manner. It is a very important activity regarding *P. aeruginosa* pathogenicity as an opportunistic bacterium. One of strategies that *P. aeruginosa* employs is producing these kinds of metabolites to sweat out the opponents (usually local natural flour) and weakening the host immune responses.

Another strategy that guarantees the survival and spread of *P. aeruginosa* is its advanced communication system, quorum sensing (QS) system that can induce biofilm or pyocyanin production, and therefore, inhibiting QS can be considered as a versatile strategy to control the infections. Here using real time-PCR, the expression of two groups of genes related to QS activity including *lasI*/*lasR* and *rhlI*/*rhlR* were measured. Gel-H.P resulted in a significant decline of the gene expression and the presence of curcumin amplified this effect. We observed slightly different activity of Gel-H.P and Gel-H.P.Cur on the QS system, which can link to HA and curcumin effects. Since biofilm production is one of the processes controlled by the QS system, the effect of HA on the inhibition of biofilm production might be related to its effect on the QS system (Bahari et al. 2017). The potential of curcumin to inhibit biofilm formation in uropathogens such as *E. coli* and *P. aeruginosa* by inhibiting the QS signaling cascade has already been identified (Packiavathy et al. 2014). Moreover, the susceptibility of uropathogens to common antimicrobials increased in the presence of curcumin (Packiavathy et al. 2014). Bahari et al. also examined the synergistic effect of curcumin with antibiotics such as azithromycin and gentamicin. In their study, the combination of sub-MIC concentrations (1/4 × MIC) of curcumin with some antibiotics significantly reduced the expression of QS regulatory genes. They emphasized that such results could lead to the development of new combination therapies against *P. aeruginosa* (Bahari et al. 2017).

**Fig. 5:**
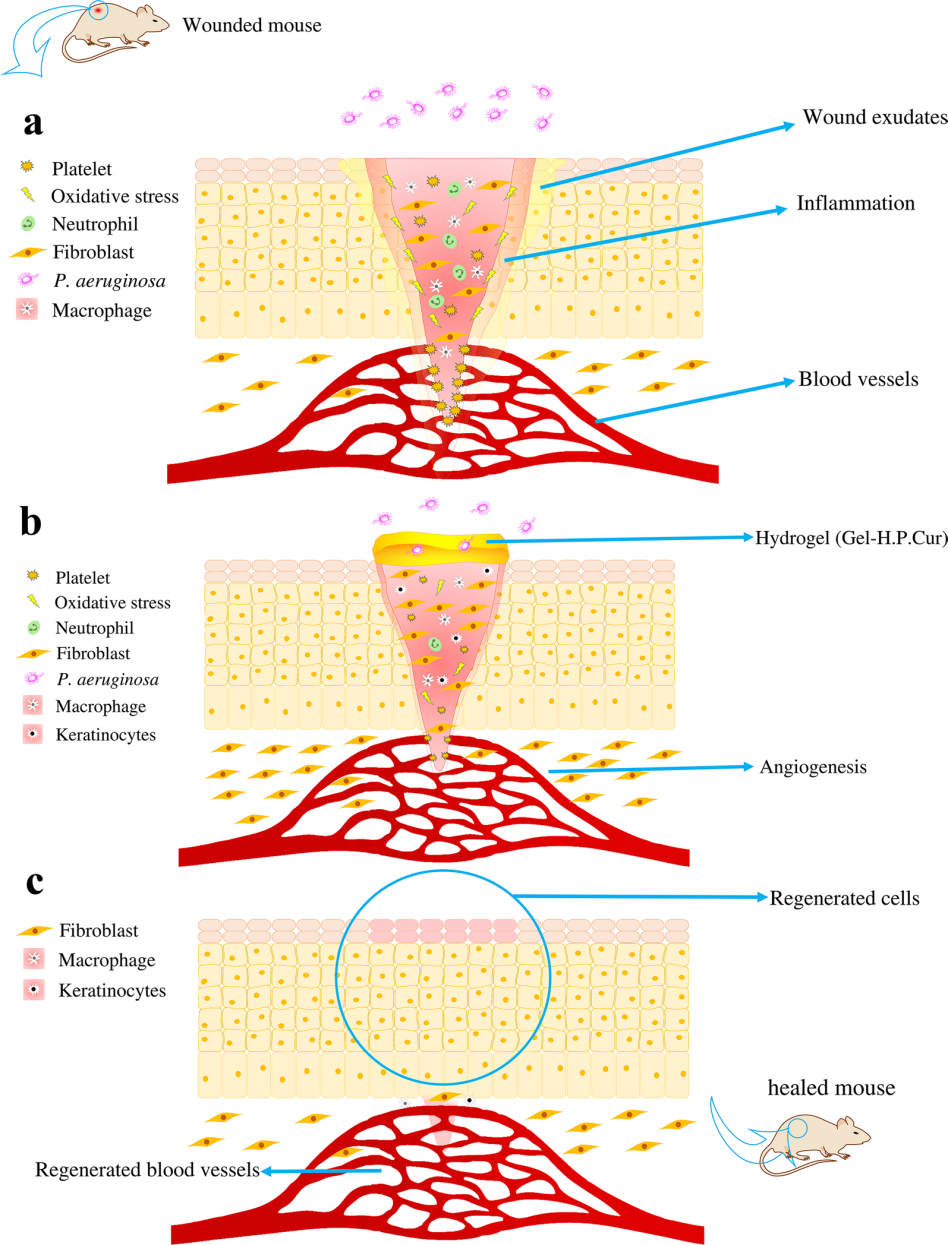
Schematic illustration of promoting wound healing process in the presence of Gel-H.P.Cur. **a** Thickness wound formation in the skin, and attacking of opportunistic bacteria. Due to the local inflammation and swollen, the wound starts to expand. **b** Treating with Gel-H.P.Cur promotes the healing processes. For instance, bacteria are prevented from entering the wound bed, and gradually the swelling and local inflammation decreases and also exudates are absorbed by the hydrogel. Proliferation of fibroblasts and angiogenesis are stimulated by both HA and curcumin. **c** After treatment with Gel-H.P.Cur the wounded tissue is completely regenerated and a slight scar is seen on the wound surface

In the last part of our study, the regenerative effect of Gel-H.P and Gel-H.P.Cur on the real skin which had infectious injury was explored. Logically, its biological role in extracellular matrix formation and maintaining, HA based biosystems should involve in regeneration of the skin damages which has been demonstrated in a number of reports (Dicker et al. 2014; Jiang et al. 2005). On the other hand, adding curcumin to Gel-H.P (Gel-H.P.Cur) improved the wound healing ability, and also rendered the high antibacterial activity. Curcumin possesses several features to make wound healing progress, such as anti-inflammatory/antioxidant activities, promoting fibroblasts migration toward the wound bed, and improving the contraction process in the wound (Akbik et al. 2014). Another advantage of using Gel-H.P.Cur in the wound healing process is leaving minimal scarring (Fig. 5). Numerous documents have shown that in infants the wound healing often completes without scarring because of a HA-enrichment ECM (Samuels and Tan 1999). This event suggests that exogenous HA can also promote wound healing process scarlessly. Although on the day 12^th^, the wound closure in the group treated with Gel-H.P.Cur (72.92% ± 3.53) (Fig. 4b, PAO1 + Gel-H.P.Cur, group 6: Table 1) is lower than that of the group treated with Gel-H.P (83.01% ± 3.51) (Fig. 4b, PAO1 + Gel-H.P., group 5: Table 1), it is worth mentioning that the extent of the wound on the day of wounding (day 0) in the first one is bigger than the second (Fig. 4a). This is while the amount of hydrogel (100 µL) was the same for all of the groups. Therefore, it is logical that a larger wound needs more time to close and heal. Also, the closure of the wound from the day 12th to the day 15th is quite relatively evident in the group treated with Gel-H.P.Cur (Fig. 4a).

Wound healing phenomenon is a complex biological process that comprises cross talks between keratinocytes, fibroblasts, and immune cells (Pastar et al. 2014). This process involves four overlapping stages including homeostasis, inflammation, proliferation, and remodeling (Clark 2013). The combination of HA and curcumin in Gel-H.P.Cur likely provided numerous healing properties. For instance, we observed that after one regime of application, the infected wound closure properly with advanced signs of skin regeneration. The studies demonstrated that HA has immunostimulatory, pro-inflammatory, and antioxidant activities (as a free radical scavenger) (Aya and Stern 2014; Jiang et al. 2011). HA also plays an efficient role in cell proliferation and angiogenesis (Frenkel 2014, Slevin et al. 2002). Angiogenesis is an essential phenomenon in the wound healing process for improving nourishment of the wound bed. Wound treatment with a healer composed of HA/Cur increases vascular endothelial growth factor (VEGF)-positive cells in the healing wound bed, as reported recently (Zhou et al. 2021).

Interestingly, in a bilateral activity, on one side HA stimulates the initiation of inflammation, and on the other side, curcumin modulates the inflammatory process. In fact, the skin-repair process is in harmony with inflammatory responses (Vikram Choudhary 2018). Nyman et al. showed that the treating of wounds with exogenous HA in the human model of deep skin lesions, accelerated re-epithelialization (Nyman et al. 2019). The role of HA in re-epithelialization may be important to avoid scarring as mentioned above (Hu et al. 2003; Mahedia et al. 2016). In addition, the antimicrobial properties of HA and curcumin can power the hydrogel to encounter infections appropriately.

Our results on the synergistic action of HA and curcumin for wound healing are consistent with recent studies. In 2021, researchers showed, the application of curcumin-loaded HA-pullulan injectable hydrogel on a rat model resulted in 90% diabetic wound closure. The authors have emphasized the role of curcumin-containing hydrogel in inhibiting inflammatory cells and enhancing tissue regeneration and angiogenesis (Shah et al. 2021).

Several studies have shown that Low molecular weight HA (LMW-HA) and high molecular weight HA (HMW-HA) can have a different effects on the expression of genes and, as a result, the physiological activity of the critical cells participate in the wound healing process (including fibroblasts and macrophages) (Maharjan et al. 2011; Rayahin et al. 2015). Although HMW-HA contributes to the clot formation process in the first moments of wounding (via binding with fibrinogen), the range of functions of LMW-HA in the wound healing process is much vaster (Huang et al. 2019). For example, it has been reported that LMW-HA has a crucial roles to elaborate the processes such as inflammatory responses, activation of macrophages, expression of chemokine and removal of free radicals (D’Agostino et al. 2015). Also, some researchers showed that LMW-HA prevents the differentiation of fibroblasts and collagen deposition in the early stages of wound healing, and consequently, induces the migration of macrophages toward the wound site with the aim of phagocytizing the cell corpses and also infectious cells (D’Agostino et al. 2015; Maharjan et al. 2011).

## Supplementary Information

**Additional file 1 :Figure S1. **Chemical structure of HA (a) and PDMS-DG (b). HA consists of repeating di-saccharide units: N-acetyl glucosamine and D-glucuronic acid. Two functional groups, hydroxyl and carboxyl, are shown by green and pink spheres. Also, an amino group can be recovered by deacetylation of the N-acetyl group (orange sphere). Polydimethylsiloxane is a kind of silicon that has two methyl groups attached to its silicon structure. PDMS-DG has two epoxy groups in its ends (blue cones). The chemical structures present here have been drawn by ChemBioDraw Ultra 12.0. **Table S1. **The primers that were used to determine the effect of Gel-H.P and Gel-H.P.Cur on the expression of QS circuit genes (lasI, lasR, rhlI and rhlR) employing real-time qPCR. **Table S2. **NMR characteristic peaks of HA, PDMS-DG and Gel-H.P

## Data Availability

All data generated or analyzed during this study are included in this published article [and its Additional file].

## Abbreviations

HA: Hyaluronic acid
PDMS-DG: Polydimethylsiloxane-diglycidyl ether terminated
Gel-H.P: Hydrogel-HA.PDMS
Gel-H.P.Cur: Hydrogel-HA.PDMS.Curcumin
QS: Quorum sensing
*P. aeruginosa*: *Pseudomonas aeruginosa*
MH: Mueller Hinton
LB: Luria Bertani
TSB: Trypticase Soy Broth
CLSI: Clinical and Laboratory Standards Institute
VEGF: Vascular endothelial growth factor
EP: Epidermis
DE: Dermis
HYP: Hypodermis
SG: Sebaceous glands
H&E: Hematoxylin and eosin
