# Supplementary material for: A fabricated hydrogel of hyaluronic acid/curcumin shows super-activity to heal the bacterial infected wound
Source: AMB Express. 2023 Mar 10;13:29. doi: 10.1186/s13568-023-01533-y (PMC10006388; doi:10.1186/s13568-023-01533-y)
Supplement: Supplementary file 1 — Additional file 1 :Figure S1. Chemical structure of HA (a) and PDMS-DG (b). HA consists of repeating di-saccharide units: N-acetyl glucosamine and D-glucuronic acid. Two functional groups, hydroxyl and carboxyl, are shown by green and pink spheres. Also, an amino group can be recovered by deacetylation of the N-acetyl group (orange sphere). Polydimethylsiloxane is a kind of silicon that has two methyl groups attached to its silicon structure. PDMS-DG has two epoxy groups in its ends (blue cones). The chemical structures present here have been drawn by ChemBioDraw Ultra 12.0. Table S1. The primers that were used to determine the effect of Gel-H.P and Gel-H.P.Cur on the expression of QS circuit genes (lasI, lasR, rhlI and rhlR) employing real-time qPCR. Table S2. NMR characteristic peaks of HA, PDMS-DG and Gel-H.P [file 13568_2023_1533_MOESM1_ESM.pdf]

A fabricated hydrogel of hyaluronic acid / curcumin shows super-activity to heal the bacterial infected wound

Maryam Khaleghi (Bioprocess Engineering Department, Institute of Industrial and Environmental Biotechnology, National Institute of Genetic Engineering and Biotechnology, Tehran, Iran. E.Mail: m_khaleghi@nigeb.ac.ir)

Fakhri Haghi* (Department of Microbiology, School of Medicine, Zanjan University of Medical Sciences, Zanjan, Iran. E.Mail: haghi@zums.ac.ir)

Mina Gholami (Department of Microbiology, School of Medicine, Zanjan University of Medical Sciences, Zanjan, Iran. E.Mail: M.Gholami137052@yahoo.com)

Hamdam Hoorfar (Bioprocess Engineering Department, Institute of Industrial and Environmental Biotechnology, National Institute of Genetic Engineering and Biotechnology, Tehran, Iran. E.Mail: Hhourfar@gmail.com)

Farshad Shahi (Bioprocess Engineering Department, Institute of Industrial and Environmental Biotechnology, National Institute of Genetic Engineering and Biotechnology, Tehran, Iran. E.Mail: F.shahi92@gmail.com)

Ali Mir Mousavi Zekoloujeh (Department of Biology, University of Zanjan, Zanjan, Iran. E.Mail: alimm689@gmail.com)

Farhang Aliakbari (Bioprocess Engineering Department, Institute of Industrial and Environmental Biotechnology, National Institute of Genetic Engineering and Biotechnology, Tehran, Iran. E.Mail: aliakbari@nigeb.ac.ir) and (Molecular Medicine Research Group, Robarts Research Institute, Schulich School of Medicine and Dentistry, University of Western Ontario, London, Ontario, Canada. E.Mail: faliakb2@uwo.ca)

Ebrahim Ahmadi (Department of Chemistry, University of Zanjan, Zanjan, Iran. E.Mail: Ahmadi@znu.ac.ir)

Dina Morshedi* (Bioprocess Engineering Department, Institute of Industrial and Environmental Biotechnology, National Institute of Genetic Engineering and Biotechnology, Tehran, Iran. E.Mail: morshedi@nigeb.ac.ir)

Corresponding authors:
Morshedi@nigeb.ac.ir (Morshedi. D.), National Institute of Genetic Engineering and Biotechnology, Address: Shahrak-e Pajoohesh, km 15 Tehran - Karaj Highway, Tehran, Iran, P.O.Box:14965/161. Phone: +9821-44878423, Fax: +9821-44878395

Haghi@zums.ac.ir (Haghi. F.), Department of Microbiology, School of Medicine, Zanjan University of Medical Sciences, Zanjan, I. R. 45139-56111, Iran

a

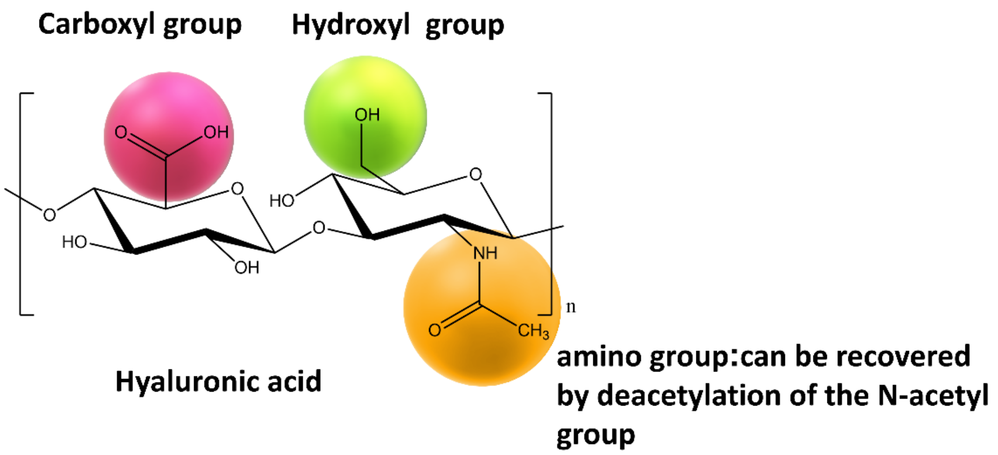

b

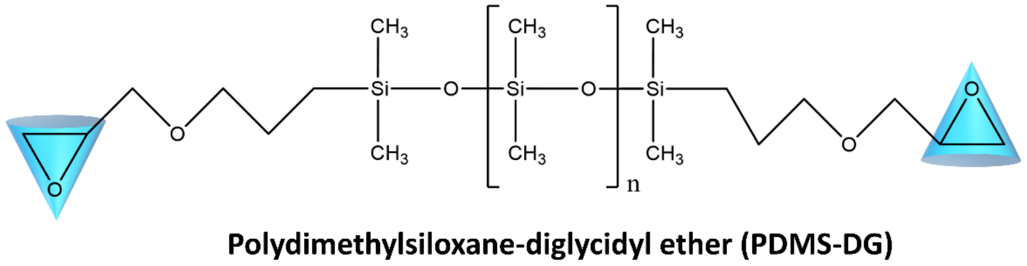

Figure S1. Chemical structure of HA (a) and PDMS-DG (b). HA consists of repeating di-saccharide units: N-acetyl glucosamine and D-glucuronic acid. Two functional groups, hydroxyl and carboxyl, are shown by green and pink spheres. Also, an amino group can be recovered by deacetylation of the N-acetyl group (orange sphere). Polydimethylsiloxane is a kind of silicon that has two methyl groups attached to its silicon structure. PDMS-DG has two epoxy groups in its ends (blue cones). The chemical structures present here have been drawn by ChemBioDraw Ultra 12.0.

Table S1. The primers that were used to determine the effect of Gel-H.P and Gel-H.P.Cur on the expression of QS circuit genes (lasI, lasR, rhlI and rhlR) employing real-time qPCR.

Gene	Primer sequence	Amplicon size (bp)
oprL	5'-AACAGCGGTGCCGTTGAC-3'<br>5'-GTCGGAGCTGTCGTACTCGAA-3'	87
lasI	5'-CGCACATCTGGGAACTCA-3'<br>5'-CGGCACGGATCATCATCT-3'	176
lasR	5'-CTGTGGATGCTCAAGGACTAC-3'<br>5'-AACTGGTCTTGCCGATGG-3'	133
rhlI	5'-GTAGCGGGTTTGCGGATG-3'<br>5'-CGGCATCAGGTCTTCATCG-3'	101
rhlR	5'-GCCAGCGTCTTGTTCGG-3'<br>5'-CGGTCTGCCTGAGCCATC-3'	160

Table S2. NMR characteristic peaks of HA, PDMS-DG and Gel-H.P

Material	Signal name	Shift (ppm)	Description
HA	1	1.9	CH3 of N-acetyl glucosamine
	2	3.502	Protons around the sugar ring
	3	3.725	Protons around the sugar ring
	4	4.673	Solvent: D_2O
PDMS-DG	a	-0.1-0.1	Si-CH_3
	b	0.501	-CH_2
	c	1.612	-CH_2
	d	2.5	Epoxy group protons
	e	2.7	Epoxy group protons
	f	3.1	Epoxy group protons
	g	3.446	-CH_2
	h	3.624	-CH_2
Gel-H.P	a	-0.1-0.1	Si-CH_3
	b	0.501	-CH_2
	c	1.612	-CH_2
	1	1.9	CH_3 of N-acetyl glucosamine
	4	4.673	Solvent: D_2O
